# A retrospective evaluation of mushroom ingestions in 421 dogs in Norway (2011–2022)

**DOI:** 10.1002/vro2.60

**Published:** 2023-04-10

**Authors:** Kristin Opdal Seljetun, Heidi Runne Kragstad

**Affiliations:** ^1^ Norwegian Poison Information Center Norwegian Institute of Public Health Oslo Norway

## Abstract

**Background:**

Mushroom poisoning may result in a variety of signs ranging from mild, mostly gastroenteritis, to organ failure and death. To increase the knowledge of prevalence, treatment and outcome in dogs, information regarding mushroom ingestion was collected.

**Materials and methods:**

This retrospective study analysed all inquiries of mushroom ingestion in dogs to the Norwegian Poison Information Center from 2011 to 2022. Mushrooms were identified by a mycologist or Norwegian‐certified mushroom expert. Differences in mushroom species, clinical findings, treatments and outcome were evaluated.

**Results:**

A total of 421 mushroom ingestions in dogs were included. The mushrooms were identified as non‐poisonous in 45% of cases. The most frequently involved toxin group was gastrointestinal mushrooms, followed by muscarinic mushrooms and mushrooms containing isoxazoles. About 64% of cases were managed at home, 33% were hospitalised and received treatment, and 3% were observed by a veterinarian without treatment. The survival rate was 98.6%, with death occurring after ingestion of *Amanita muscaria*, *Cortinarius rubellus*, *Amanita virosa*, *Clitocybe rivulosa* and *Inocybe* sp.

**Conclusions:**

This study demonstrated the importance of rapid and accurate identification of the mushroom. This could prevent delays in therapeutic intervention and avoid unnecessary treatment of these dogs. With early, correct identification of mushrooms, our results demonstrated a good prognosis for dogs after ingestion.

## INTRODUCTION

The number of mushroom species found throughout the world has been estimated to be over 5000; fortunately, only 3% of these are poisonous.^1^ Mushroom ingestion by dogs is not uncommon because of their indiscriminate eating habits. There are few reports of poisoning of dogs by mushrooms with confirmed identification.

Depending on the species, ingestion of mushrooms can result in various signs from no toxic effect to mild gastrointestinal (GI) disturbances and, in severe cases, organ failure or death.^2^ Identifying mushroom species can be complex, and a lack of mushroom material is often a limiting factor for accurate diagnosis and treatment.

The objective of a poison centre is to assess toxicity and, if necessary, provide information about first aid to the general public to reduce the time between suspected ingestion of mushrooms and hospitalisation and to provide treatment advice for health professionals. The Norwegian Poisons Information Centre (NPIC) is a nationwide, free, 24‐h access telephone service for the general public, human health professionals and veterinarians. Some of the staff at the NPIC are Norwegian‐certified mushroom experts. A consultant service including a professional mycologist is provided. The mycologist has expert knowledge in identifying mushrooms by photographs sent by mobile telephone together with a description of the habitat and geographical area. The consultant is contracted to reply to the call within 10 min of receiving the images and provides follow‐up calls when deemed necessary. Biological material is analysed microscopically when needed.

To the best of the authors’ knowledge, there has been no comprehensive review of mushroom ingestion in dogs with identification performed by a mushroom specialist in Norway. The aim of this study was to retrospectively evaluate the clinical details, treatment and outcome in a population of dogs after mushroom ingestion. Severe poisoning from any type of mushroom was analysed in detail.

## MATERIALS AND METHODS

All ingestions of mushrooms by dogs with a confirmed identification by a mycologist or Norwegian‐certified mushroom expert contacting the NPIC from January 2011 to December 2022 were included. Case data, including species, breed, age, weight, sex, estimated dose and time of ingestion, were collected at contact. Follow‐up data regarding onset, severity and duration of clinical signs, possible treatment and long‐term outcome were obtained by a telephone call to veterinarians and/or dog owners. All abnormalities noted by the owner and/or veterinarian were recorded.

Dogs were identified as juvenile if they were under 1 year of age, adults between the ages of 1 and 9 years and geriatric if they were older than 10 years of age.

The amount ingested was classified as lick (in mouth, but entire mushroom removed), a bite, one mushroom, multiple mushrooms or uncertain (swallowed, but uncertain amount).

Dogs were included in the study if mushroom ingestion was witnessed. The mushroom had to be identified by a mycologist or Norwegian‐certified mushroom expert. Exclusion criteria included lack of identification, minimal amount ingested (including ‘lick’ amount), unavailable follow‐up information, incomplete medical records or other animal species.

The severity of the clinical signs was classified as none, minor, moderate, severe and fatal, utilising a published human poisoning severity score and then adapted as an approximation for use in dogs.^3^

Mushrooms were grouped by toxicity into three groups as edible, non‐edible and poisonous. Poisonous mushrooms were classified by toxins into eight groups, including muscarine, amatoxin, isoxazoles, orellanine, coprine, gyromitrin, psilocybin and GI irritants.

## RESULTS

From January 2011 to December 2022, there were 672 calls to the NPIC for potential mushroom ingestion in animals; 421 cases met the inclusion criteria and were evaluated, while 251 cases were excluded. The reasons for exclusion were lack of identification (68), uncertain ingestion or minimal amount ingested (86), follow‐up information unavailable (80), incomplete medical records (3) and other animal species (14). Calls about other animal species with mushroom ingestion included cat (10), heifer (2), horse (1) and sheep (1).

The majority of the 421 calls (79.3%) came from dog owners, while veterinarians contacted the NPIC in 20.7% of cases.

Female dogs were slightly overrepresented (44.9%) when compared to male dogs (39.4%). The sex of 66 cases (15.7%) was not recorded. The median age of the dogs in this study was 5 months (1.5–180 months). Most cases involved juvenile dogs (72.4%). Adult dogs comprised 24.5% and geriatric dogs 2.1% of cases, while the age of four dogs (1%) was not recorded.

A total of 99 dog breeds were reported, with the most commonly affected breed being mixed breed, followed by golden retriever and Labrador retriever (Table 1). Seven cases did not have breeds recorded. The median weight of all dogs was 7.5 kg (range 0.7–51 kg). Bodyweight was not recorded for 16 dogs.

**TABLE 1:** Most common breeds with reported mushroom ingestion (*n* = 421).

Breed	Number of dogs	Percentage of cases
Mixed breed	67	15.9
Golden retriever	20	4.8
Labrador retriever	20	4.8
Whippet	12	2.9
Cocker spaniel	11	2.6

The cases were analysed for seasonality of presentation, including January (2; 0.5%), February (2; 0.5%), March (0; 0%), April (5; 1.2%), May (25; 5.9%), June (33; 7.8%), July (51; 12.1%), August (74; 17.6%), September (126; 29.9%), October (86; 20.4%), November (14; 3.3%) and December (3; 0.7%). The mean numbers of cases in August, September and October were 6.7, 11.5 and 7.8, respectively, which is the main wild mushroom season in Norway.

The majority of dogs ingested mushrooms growing in the garden (61.5%), mushrooms in the forest or woodland were involved in 20.9% of cases, while 5.9% of the dogs ingested mushrooms growing in the mountains. The location was documented as other in 8.3% of cases and not recorded in 3.3% of cases.

A total of 271 (64.4%) of cases were managed at home, 139 (33.0%) were hospitalised and received treatment, and 11 (2.6%) were observed by a veterinarian without treatment. The survival rate was 98.6%, with death occurring after ingestion of *Amanita muscaria* (*n* = 2), *Cortinarius rubellus* (*n* = 1), *Amanita virosa* (*n* = 1), *Clitocybe rivulosa* (*n* = 1) and *Inocybe* sp. (*n* = 1).

A total of 121 mushroom species were included in the study. The most frequently involved toxin groups were GI mushrooms (28.3%), muscarinic mushrooms (12.8%) and mushrooms containing isoxazoles (4.0%) (Table 2).

**TABLE 2:** Numbers of dogs with mushroom poisoning by toxin group (*n* = 421).

Toxin group	Number of dogs (%)	Mushrooms included
Gastrointestinal irritants	119 (28.3%)	Various
Muscarine	54 (12.8%)	*Clitocybe ribulosa*, *Inocybe geophylla*, *Inocybe rimosa*, *Inocybe dulcamara*, *Inocybe lacera*, *Inocybe lanuginosa*, *Inocybe* sp.
Isoxazoles	17 (4.0%)	*Amanita muscaria*, *Amanita pantherina*, *Amanita regalis*
Coprine	16 (3.8%)	*Coprinellus micaceus*, *Coprinopsis atramentaria*, *Coprinellus* sp.
Psilocybin	13 (3.1%)	*Panaeolina foenisecii*
Hydrazines	10 (2.4%)	*Gyromitra esculenta*
Amatoxin	3 (0.7%)	*Amanita virosa*
Orellanine	1 (0.2%)	*Cortinarius rubellus*

### Muscarine

Fifty‐four dogs (12.8%) were included after ingestion of muscarinic mushrooms. Seven of these mushrooms were identified microscopically. *C. rivulosa* was ingested in eight cases (four mild, three moderate and one fatal), *Inocybe geophylla* (*n* = 3; two mild, one moderate) and *Inocybe rimosa* accounted for two poisonings (both moderate). *Inocybe dulcamara* (*n* = 1), *Inocybe lacera* (*n* = 1) and *Inocybe lanuginosa* (*n* = 1) resulted in two mild and one moderate poisonings. In the remaining 37 cases (13 mild, 20 moderate, three severe and one fatal), the mushrooms were identified as genus *Inocybe*, although the exact species could not be determined.

Forty‐five (83.3%) of the 54 muscarinic mushrooms were growing in the garden, while 7.4% were growing in the forest. In four cases, the location was other or unknown. The amount ingested was a bite in 37.0% (20/54) of cases, while 24.1% (13/54) ingested an entire mushroom, 16.7% (9/54) ingested multiple mushrooms and 22.2% (12/54) ingested an uncertain amount.

All the 54 dogs developed clinical signs. The latency between ingestion and onset of signs ranged from 5 to 120 min. The most common signs were hypersalivation (100%), vomiting (81.5%), diarrhoea (79.6%) and lethargy (68.5%) (Table 3).

**TABLE 3:** Clinical signs associated in dogs with muscarinic mushroom (*n* = 54) and isoxazoles mushroom (*n* = 17) ingestion.

Clinical sign	Number of dogs	Percentage
**Muscarinic mushrooms**		
Hypersalivation	54	100
Vomiting	44	81.5
Diarrhoea	43	79.6
Lethargy	37	68.5
Urination	10	18.5
Tremor	9	16.7
Abdominal pain	6	11.1
Lacrimation	6	11.1
Restlessness	6	11.1
Bradycardia	5	9.3
Dyspnoea	5	9.3
Ataxia	5	9.3
Miosis	4	7.4
Rhinorrhoea	4	7.4
Hypothermia	3	5.6
Pale mucous membranes	3	5.6
Tachycardia	3	5.6
Tachypnoea	2	3.7
Hypotension	2	3.7
Haematemesis	1	1.9
**Isoxazoles mushrooms**		
Lethargy	6	35.3
Vomiting	5	29.4
Tremor	5	29.4
Hypersalivation	4	23.5
Ataxia	4	23.5
Seizures	4	23.5
Behavioural abnormalities	3	17.6
Diarrhoea	3	17.6
Restlessness	3	17.6
Tachypnoea	2	11.8
Tachycardia	2	11.8
Nystagmus	1	5.9
Abdominal pain	1	5.9
Dyspnoea	1	5.9
Miosis	1	5.9

Forty‐four (81.5%) of the 54 dogs that ingested muscarinic mushrooms were hospitalised. Treatment in veterinary clinics/hospitals included emetic 22.7% (10/44), activated charcoal 36.4% (16/44), multiple‐dose activated charcoal 20.5% (9/44), intravenous fluids 70.5% (31/44), maropitant 31.8% (14/44), atropine 29.5% (13/44), probiotics 27.3% (12/44), methadone 4.5% (2/44), sucralfate 4.5% (2/44), antibiotics 2.3% (1/44) and metoclopramide 2.3% (1/44). Except for the two fatal cases, all dogs recovered without sequela. The duration of clinical signs in the survivors ranged from 20 min to 3 days (mean 14.7 h). In four dogs, duration of clinical signs was not reported.

### Isoxazoles

Seventeen dogs (4.0%) ingested mushrooms with isoxazole toxins divided between *A. muscaria* (*n* = 11), *Amanita pantherina* (*n* = 3) and *Amanita regalis* (*n* = 3).

Clinical signs were seen in 10 dogs (58.8%). In all seven subclinical cases, the dog ingested a bite. Three of these were administered an emetic at a veterinary clinic, while two were given activated charcoal. Of the 10 dogs showing clinical signs, one developed mild signs, five moderate signs, two severe signs and two were fatal signs. The two fatal cases died within 2 h of ingestion before reaching veterinary hospital after one and multiple *A. muscaria* mushrooms, respectively. Hence, 52.9% of the ingestions (9/17) resulted in moderate to severe poisoning. Eleven of the 17 dogs ingesting isoxazole mushrooms were hospitalised. Treatment at veterinary clinic/hospital included emetic (6/11), activated charcoal (9/11), multiple‐dose activated charcoal (3/11), intravenous fluids (5/11), sedative (2/11), maropitant (1/11) and glucose (1/11).

The latency between ingestion and onset of signs ranged from 20 min to 4 h (mean 1.7 h). In the clinically affected dogs, 90% (9/10) showed CNS signs with lethargy, hypersalivation, ataxia, restlessness, miosis, behavioural abnormalities, tremors or seizures (Table 3). Gastrointestinal signs were seen in 60% (6/10) with vomiting, diarrhoea or abdominal pain. The duration of clinical signs in the survivors ranged from 20 h to 2 days (mean 30.6 h).

### Amatoxin

Three dogs in this study ingested *A. virosa*. In all three cases, the amount ingested was determined to be a bite. An 18 kg English springer spaniel vomited 8–9 h after ingestion of approximately a tablespoon of *A. virosa*. Furthermore, profuse, watery diarrhoea and abdominal pain were reported. The dog was brought to a veterinary clinic 26 h after ingestion recumbent with abdominal pain, hyperthermia (40°C), tachycardia, dilated pupils, weak pulse and acute hepatitis. Symptomatic treatment was initiated and the dog died 22 h after initial presentation. The remaining two dogs in the study were given an emetic shortly after ingestion, producing mushroom remnants in the vomit. Further treatment consisted of activated charcoal and intravenous fluids. One of the dogs received acetylcysteine. No clinical signs of poisoning were observed in these dogs.

### Orellanine

One dog, 6.6 kg, ingested parts of one *C. rubellus* containing the nephrotoxic compound orellanine. Three days after ingestion, the dog developed clinical signs with repeated vomiting (×5), abdominal pain and lethargy. The recumbent dog was hospitalised at day 4. Urine specific gravity was 1.011 and blood analysis displayed azotaemia, hyperphosphatemia, hyperglycaemia and hyponatremia, together with increase in albumin and globulin consistent with mild dehydration. Treatment consisted of continuous intravenous fluids, maropitant and buprenorphine. Despite treatment, the dog remained obtunded with deteriorating clinical condition. Acute kidney injury (AKI) was diagnosed based on progressive increases in serum creatinine and blood urea nitrogen. The dog was euthanased at its owners’ request day 5 after ingestion due to severe AKI (grade IV), increasing pancreatic enzymes and poor prognosis.

### Gyromitrin

*Gyromitra esculenta* was ingested by 10 dogs. The amount ingested was a bite (*n* = 2) or unknown (*n* = 8). Vomiting (*n* = 4) and diarrhoea (*n* = 1) were seen in five cases. All dogs were treated by a veterinarian with emetic (*n* = 10), activated charcoal (*n* = 8) and probiotics (*n* = 1) with no further clinical signs reported.

### Coprine

Sixteen dogs ingested coprine‐containing mushrooms: *Coprinellus micaceus* (*n* = 5), *Coprinopsis atramentaria* (*n* = 10) and *Coprinellus* sp. (*n* = 1). No clinical signs were seen in 10 of the cases, while six dogs developed mild signs. Clinical signs consisted of vomiting (*n* = 3), diarrhoea (*n* = 3), lethargy (*n* = 1) or hypersalivation (*n* = 1). Two cases were given an emetic at a veterinary clinic and the remaining cases were observed at home without treatment.

### Psilocybin

*Panaeolina foenisecii* was ingested by 12 dogs. The amount was determined to be a bite (*n* = 7), one mushroom (*n* = 3) or unknown (*n* = 1), while one dog ingested two to three mushrooms. Nine dogs developed no signs, while mild poisoning was seen in three cases. Clinical signs in the mild poisoning cases consisted of diarrhoea (*n* = 2), vomiting (*n* = 1), anorexia (*n* = 1) or lethargy (*n* = 1).

### Gastrointestinal irritants

Of the 421 dogs, 119 ingested GI irritant mushrooms (28.3%). Of these 61.3% (73/119) remained asymptomatic, while 35.3% (42/119) developed signs consistent with mild poisoning. Moderate poisoning was observed in 2.5% (3/119) of cases and severe poisoning was observed in 0.8% (1/119) of cases.

Clinical signs were seen from 15 min to several hours after ingestion and consisted of vomiting (36/46, 78.3%), diarrhoea (23/46, 50.0%), lethargy (13/46, 28.3%), hypersalivation (8/46, 17.4%), anorexia (3/46, 6.5%), ataxia (2/46, 4.3%), hypothermia (2/46, 4.3%), haematochezia (2/46, 4.3%), tremor (1/46, 2.2%), bradycardia (1/46, 2.2%) and tachycardia (1/46, 2.2%).

Forty‐four of the 119 cases received veterinary treatment, including emetic (28/44, 63.6%), activated charcoal (17/44, 38.6%), intravenous fluids (15/44, 34.1%), probiotics (10/44, 22.7%), antiemetic (3/44, 6.8%) or sucralfate (1/44, 2.3%).

All dogs recovered without complications within 2 days.

### Other mushrooms

In 189 cases (44.9%), the mushrooms were identified as non‐poisonous: edible (*n* = 106), non‐edible (*n* = 74) and spoiled (*n* = 9), with clinical signs reported in 48 dogs. Ingestion of edible mushrooms resulted in clinical signs in 23 cases with GI disturbances (vomiting 14/106, diarrhoea 11/106, hypersalivation 2/106, anorexia 3/106) and other mild signs (lethargy 6/106, ataxia 3/106, tremor 2/106). Similar signs were seen in 18 cases after ingestion of non‐edible mushrooms (vomiting 13/74, diarrhoea 11/74, hypersalivation 5/74, lethargy 4/74). Spoiled mushrooms resulted in vomiting in three of the nine cases. Hence, non‐poisonous mushrooms caused GI disturbances in 43 cases (22.8%). In these cases, the amount ingested was determined to be a bite (*n* = 25), one mushroom (*n* = 5) or uncertain (*n* = 6), while seven dogs ingested several mushrooms. In two cases dogs played with *Bovista* sp. resulting in self‐limiting coughing, lasting 1 h.

## DISCUSSION

This study presents the clinical course and outcome for 421 dogs with confirmed mushroom ingestion, identified by a mycologist or Norwegian‐certified mushroom expert.

A variety of dog breeds ingested mushrooms in our study. Mixed breed dogs, Labrador retriever and Golden retriever were the most commonly presented breeds. This is likely a result of the popularity of these breeds rather than a true breed predisposition.

Muscarinic syndrome was the most common poisoning in the dogs ingesting mushrooms in our study. The high prevalence of *Clitocybe* sp. or *Inocybe* sp. could be due to their prevalence and widespread growth in grasslands, lawns and roadsides where dogs are commonly frequent. This is reflected in our numbers, with 83.3% of the muscarinic mushrooms ingested from the garden. Similar to previous reports, all the dogs ingesting these species developed clinical signs a short time after ingestion (from 5 to 120 min).^4^, ^5^, ^6^, ^7^ The most common clinical signs were hypersalivation (100%), vomiting (81.5%), diarrhoea (79.6%) and lethargy (68.5%). Tachycardia was observed in three dogs, although bradycardia is the common clinical sign in muscarinic poisoning. However, tachycardia could be caused by fluid loss because these dogs presented with profuse hypersalivation, diarrhoea and vomiting.^8^ Atropine is the recommended treatment for muscarinic poisoning and resulted in rapid improvement in the 13 dogs treated in our study. Eleven of the dogs (27.2%) were treated with probiotics. This is generally considered a safe treatment. However, the effect of treatment for acute GI disease is probably limited and there is currently no indication of probiotic therapy for muscarinic poisoning.^9^

To the best of the authors’ knowledge, we have documented the first case of accidental orellanine poisoning in a dog. Previously, severe renal damage has been reported in four sheep grazing a field where *C. rubellus* was present.^10^ Experimental poisoning in cats resulted in acute tubular necrosis consistent with the histopathological changes found in humans after accidental poisoning.^11^, ^12^ The dog in our study displayed signs 3 days after ingestion, which is consistent with severe orellanine poisoning in humans and a previous experiment in dogs.^13^, ^14^

The ingestion of *Amanita phalloides* has previously been reported to result in severe poisoning and death in dogs.^15^ However, this species is less common in Norway; hence, no cases were identified in our study. One dog ingested *A. virosa*, resulting in vomiting, diarrhoea, abdominal pain, recumbency, hyperthermia, acute hepatic necrosis and death, similar to reports of ingestion of other *Amanita* species.^15^, ^16^ However, our results demonstrate favourable results of rapid decontamination and supportive treatment in two cases.

Several dogs in our study ingested *Amanita* species containing isoxazoles. The latency between ingestion and the onset of signs is reported to be between 0.5 and 2 h of ingestion.^2^ This is in accordance with our findings, although in one case, the latency was unusually long (4 h). The toxicity of isoxazoles is not well documented in dogs; our results demonstrated a high toxicity with moderate to severe poisoning in 52.9% of cases. Two dogs died within 2 h after ingestion of one and multiple *A. muscaria*, respectively, corresponding to a previous report.^17^ In the clinically affected dogs, CNS signs (90%) and GI distress (60%) were common, in accordance with previously published cases.^17^, ^18^, ^19^ Decontamination is recommended in asymptomatic dogs, followed by supportive treatment. Caution is recommended when treating with GABAergic medications such as benzodiazepines because muscimol and ibotenic acid bind to GABA receptors and can exacerbate CNS and respiratory depression.^2^

Multiple doses of activated charcoal were administered in some cases after ingestion of muscarinic mushrooms (*n* = 9) and isoxazoles (*n* = 3). In several cases, treatment was initiated before contacting the NPIC. Furthermore, the use of multiple doses of activated charcoal in these cases could be due to inexperience by the veterinarian in the treatment of mushroom toxicosis.

Ingestion of *G. esculenta* previously resulted in fatal haemolytic crisis in a dog.^20^ In our study, the ingested amount was determined to be small, with one bite in two cases and unknown in the additional eight cases. The minor ingestions and rapid decontaminations could explain lack of significant signs in the dogs, although mild GI signs were seen in five cases.

*Panaeolina foenisecii* is suspected to contain psilocybin, although chemical analyses have demonstrated varying results.^21^, ^22^ The 13 dogs in our study ingesting this mushroom did not display any clinical signs coinciding with psilocybin poisoning, similar to a study with accidental ingestion in children.^23^ However, as no chemical analyses were performed in our study, the hallucinogenic properties of *P. foenisecii* cannot be determined.

In 189 cases, the dogs ingested non‐poisonous mushrooms, which are unlikely to cause signs of toxicity. However, GI disturbances were observed in 22.8% of these dogs. Dogs are indiscriminate eaters and mushrooms are hard to digest.^24^ Nevertheless, 58.1% of the dogs exhibiting GI signs ingested only a bite of the mushroom. Furthermore, GI disturbances can be caused by malabsorption of proteins and sugars or microorganisms infecting the mushrooms.^25^ However, only 13 of these dogs needed veterinary treatment.

This retrospective study has several inherent limitations. First, the retrospective nature of the study prevented uniform data collection for all cases. Additionally, different time points at which the dog developed clinical signs were not known for all dogs, making the exact time of onset and duration of clinical signs difficult to derive. Further limitation is that the exact amount of mushroom ingested could not be verified and was based on estimation from the dog owner. Nevertheless, in most cases, the amount missing from the remaining mushroom gave a good estimation of the ingested dose. However, this prevented correlation of ingested amounts with the development of clinical signs in the dogs. Finally, due to limited sample size of each species and numerous veterinarians and clinics involved, a standardised treatment plan was not applied. Hence, the individual treatments could not be evaluated separately. Although the limited sample size of each species in this study does not allow for statistical analyses, the descriptive information obtained is clinically relevant to increase knowledge to owners and veterinarians about mushroom toxicosis in dogs. The vast majority (64.4%) of mushrooms ingested by dogs in our study were harmless and they could be observed at home after identification. This demonstrates the importance of early identification by a mycologist to provide appropriate diagnosis and treatment advice in dogs ingesting unknown mushrooms. Furthermore, our results emphasise that prompt veterinary treatment can result in a successful outcome even after ingestion of severely toxic mushrooms.

This study demonstrated the importance of rapid and accurate identification of the mushroom. In cases where the mushroom is retrieved and identification is possible, this will prevent delay in therapeutic intervention and avoid unnecessary treatment of these dogs. About 64% of dogs in the present study were observed at home without the need for veterinary consultation and the survival rate was 98.6%. The results suggest a good prognosis for dogs after ingestion and early identification of mushrooms.

## AUTHOR CONTRIBUTIONS

Kristin Opdal Seljetun was responsible for the study design, data collection, analysis, drafting, writing and editing of the manuscript. Heidi Runne Kragstad was responsible for data collection, analysis, writing and editing the manuscript.

## CONFLICTS OF INTEREST STATEMENT

The authors declare they have no conflicts of interest.

## FUNDING INFORMATION

The authors received no specific funding for this work.

## ETHICS STATEMENT

No ethical approval for this study was required because this was a retrospective review.

## Data Availability

The data that support the findings of this study are available from the corresponding author upon reasonable request.
